# Use of oral gadobenate dimeglumine to visualise the oesophagus during magnetic resonance angiography in patients with atrial fibrillation prior to catheter ablation

**DOI:** 10.1186/1532-429X-16-41

**Published:** 2014-06-13

**Authors:** Riccardo Faletti, Alessandro Rapellino, Francesca Barisone, Matteo Anselmino, Federico Ferraris, Paolo Fonio, Fiorenzo Gaita, Giovanni Gandini

**Affiliations:** 1 Istituto di Radiologia dell’Università degli Studi di Torino, A.O. Città della Salute e della Scienza, Via Genova 3, 10126 Torino, Italy; 2 Istituto di Cardiologia dell’Università degli Studi di Torino, A.O. Città della Salute e della Scienza, Corso Dogliotti 14, 10126 Torino, Italy

**Keywords:** Atrial fibrillation, Percutaneous endocardial radiofrequency ablation, Magnetic resonance angiography, Atrio-esophageal fistula, Gadobenate dimeglumine oral administration

## Abstract

**Background:**

Atrio-oesophageal fistula was first reported as a fatal complication of surgical endocardial and percutaneous endocardial radiofrequency ablation for atrial fibrillation, with an incidence after catheter ablation between 0.03% and 0.5%. Magnetic resonance angiography (MRA) was usually performed to obtain pre-procedural 3D images, used to merging into an electro-anatomical map, guiding step-by-step ablation strategy of AF. Our aim was to find an easy, safe and cost-effective way to enhance the oesophagus during MRA.

**Methods:**

In 105 consecutive patients, a right-left phase encoding, free breathing, 3D T1 MRA sequence was performed in the axial plane, >24 hours before catheter ablation, using an intravenous injection of gadobenate dimeglumine contrast medium. The oesophagus was enhanced using an oral gel solution of 0.7 mL gadobenate dimeglumine contrast medium mixed with approximately 40 mg thickened water gel, which was swallowed by the patients on the scanning table, immediately before the MRA sequence acquisition.

**Results:**

The visualisation of the oesophagus was obtained in 104/105 patients and images were successfully merged, as left atrium and pulmonary veins, into an electro-anatomical map, during percutaneous endocardial radiofrequency ablation. All patients tolerated the study protocol and no immediate or late complication was observed with the oral contrast agent administration. The free-breathing MRA sequence used in our protocol took 7 seconds longer than MRA breath-hold conventional sequence.

**Conclusion:**

Oesophagus visualization with oral gadobenate dimeglumine is feasible for integration of oesophagus anatomy images into the electro-anatomical map during AF ablation, without undesirable side effects and without significantly increasing cost or examination time.

## Background

Atrio-oesophageal fistulae were first reported as a fatal complication of endocardial surgical radiofrequency (RF) ablation for atrial fibrillation (AF) [1], and have since been reported after percutaneous endocardial RF catheter ablation (RFCA) [2,3]. The incidence of atrio-oesophageal fistula after catheter ablation was estimated at 0.03–0.5% [4,5] and associated with high mortality rates [6], even when the correct diagnosis was made relatively early in the clinical course. Atrio-oesophageal fistula after catheter ablation is caused by heat transfer to the oesophagus [7], which causes transmural tissue necrosis [8], mediastinitis, and a fistulous connection between the oesophageal lumen and the left atrial blood pool. Thus, prevention of the initial oesophageal injury during the ablation procedure is important if the development of atrio-oesophageal fistula is to be avoided.

Any strategy to limit or avoid RF energy delivery in close proximity to the oesophagus requires that the clinician have accurate information regarding the anatomy since the oesophagus is anatomically close to the left atrium (LA). However, the anatomical relationship between the oesophagus and the LA is highly variable [9,10]. In 15% of patients, minor oesophageal lesions have been detected as ulcerations by endoscopy [11]. Moreover, cardiac magnetic resonance angiography (MRA) or computed tomography (CT)-angiography (CTA) are often performed to obtain pre-procedural three-dimensional (3D) images of the anatomy of the LA and pulmonary veins (PVs) before the RFCA procedure. The electro-anatomical map was then integrated with 3D images of MRA or CTA to create a map that provides a step-by-step ablation strategy for AF.

The purpose of this study was to assess oesophageal anatomy and position prior to MRA using a technique that integrates an electro-anatomical map during the RFCA procedure.

## Methods

This study included 105 consecutive patients with AF (92 males, 13 females, mean age 55.2 years) that were candidates for RFCA (Table 1). Three patients suffered from mild achalasia due to neurological complications. In all patients MRA was performed within 24 h before catheter ablation using gadobenate dimeglumine contrast medium (Gd-Bopta, Multihance, Bracco Altana Pharma, Constance, Germany) for intravenous and oral administration. We collected informed consent for the MR examination and for oral administration of the contrast medium and our study design followed Declaration of Helsinki recommendations. After the MR examination, all patients were clinically monitored for 30 min before being returned to the cardiology department. Any adverse effects or anomalies were registered after RCFA prior to discharge.

**Table 1:** Patients characteristics

	**Men**	**Women**
Number of Patients	92	13
Age (years)	58,5 (42 to 70)	52,6 (39 to 69)
Paroxistic Atrial Fibrillation	70 (76%)	9 (70%)
Persitent Atrial Fibrillation	22 (24%)	4 (30%)
Duration of Atrial Fibrillation (years)	6.4	6.1

### MRA study protocol

We used a 1.5-Tesla Magnet (Philips Medical Systems, DA Best, Netherlands) with a cardiac coil volumetric phased array consisting of 32 elements from a rigid rear portion and a flexible front, each consisting of a 16-channel phased array. MRA was performed in the axial plane with a non-ECG-gated, breath-free sequence, Spoiled Gradient Eco 3D (TR 3–8 ms, TE 1–3 ms), with R-L phase encoding, using a FOV of 450 mm, 512 × 512 matrix with an isotropic voxel of 1.5 mm and flip angle of 20°. The mean sequence time was 25 s (range, 19–32 s). The acquisition was performed after intravenous infusion of gadobenate dimeglumine contrast agent (infusion rate = 2.5 ml/sec; 0,05 mmol*kg), followed by a 20-ml saline bolus. Bolus tracking was used to start the sequence at the exact moment at which the contrast intensified during the venous phase of pulmonary circulation to guarantee the maximum signal intensity in the PVs and into the LA.

The oesophagus was intensified by administration of a 0.7–0.8-ml gel solution of gadobenate dimeglumine contrast media mixed with ~40-mg thickened water gel (Nestlé Healthcare Nutrition SA, Barcelona, Spain), served with a disposable plastic spoon, while the patients were on the scanning table immediately before MRA sequence acquisition. The patients were previously instructed to swallow the bolus 2–3 s before starting the MRA sequence. A second-pass MRA sequence was acquired immediately after with the same first-pass settings to obtain suitable images in case of initial technical problems. During post-processing, LA volume and left appendage volume were calculated by locating the regions of interest, while 3D MIP and 3D volume rendering reconstruction was performed to assess the spatial position of the oesophagus and identify appendage morphology and any PV anatomical variations.

## Results

**Figure 1:**
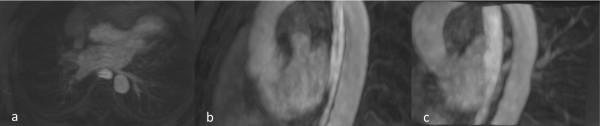
**MRA of a 66 years old male with AF.** MIP reconstruction in post-processing of MRA images acquired using both venous and oral Gadobenate Dimeglumine, 24 hr before percutaneous catheter ablation: **(a)** Axial MIP reconstruction **(b)** Left sagittal 3D MIP reconstruction **(c)** Left-Posterior 3D MIP reconstruction.

**Figure 2:**
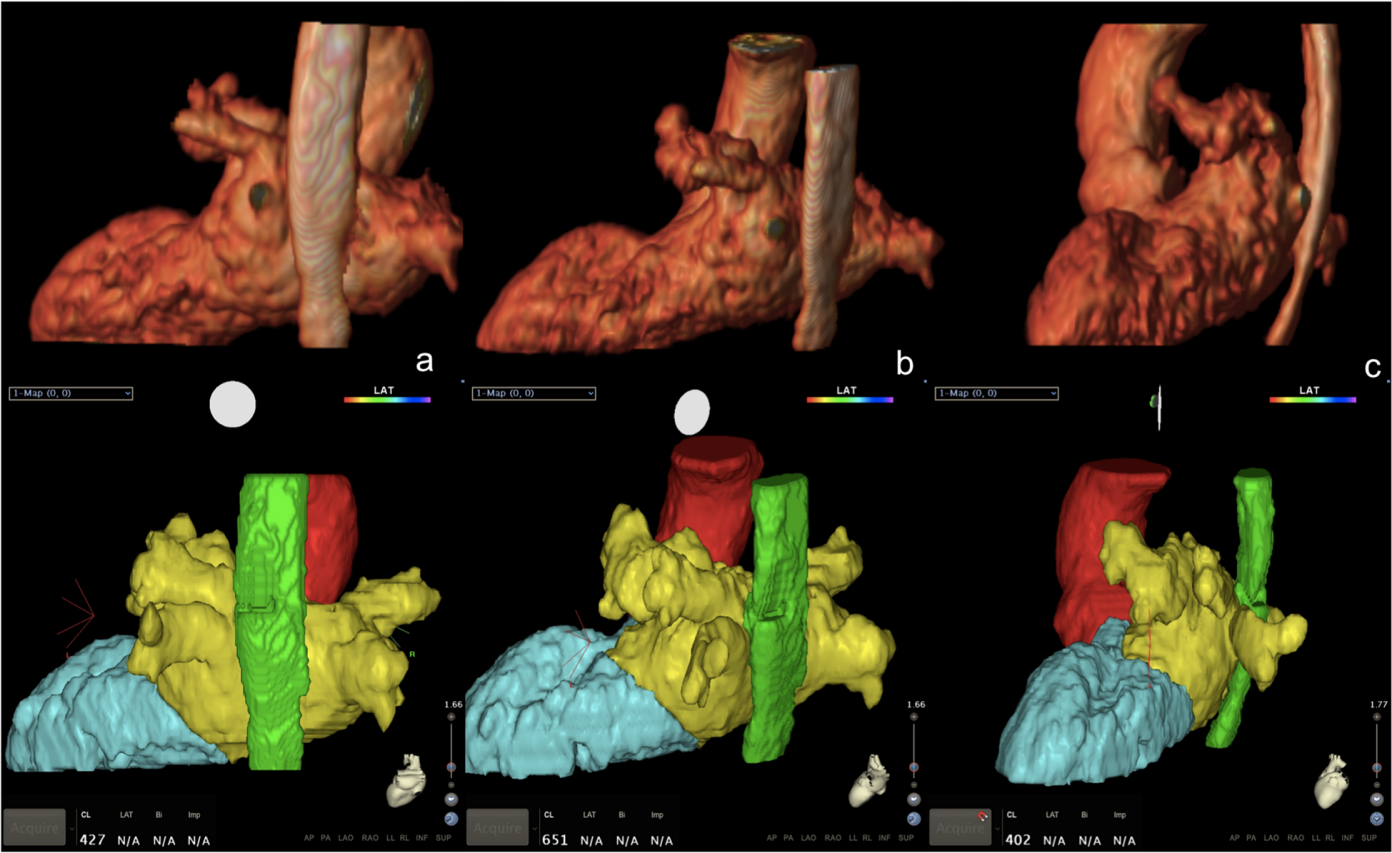
**Same patient.** MRA images and Electro-anatomical map 3D merging. On top line: 3D volume rendering from 3D GRE T1 scan; on bottom line: CARTO-MERGE^tm^ map using MRA examination data during RFCA procedure; **(a)** Posterior Coronal **(b)** Left-Posterior oblique **(c)** Left Sagittal.

**Figure 3:**
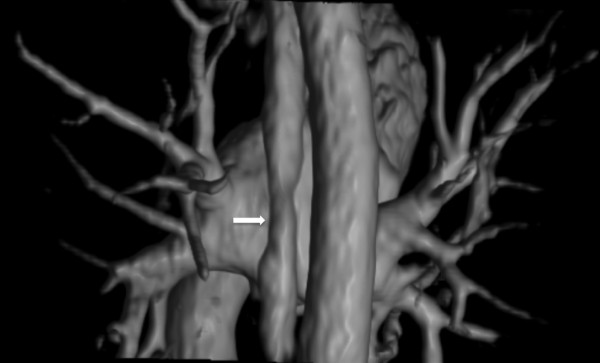
**MRA of a 56 years old male, suffering AF.** 3D volume rendering from 3D GRE T1 scan, showing the incomplete filling of the oesophagus (white arrow), due to a peristaltic wave.

Our technique was tested initially on a healthy volunteer to demonstrate feasibility and predict problems during the MR examination following gel administration. During the study, all patients tolerated the study protocol and no immediate or late complication was observed after administration of the oral contrast agent. The oesophagus was visualised and successfully merged into the electro-anatomical maps in 104/105 patients (Figure 1; Figure 2). One patient did not swallow the bolus because he did not hear our instruction to do so. We obtained a fully enhanced oesophagus in 100/104 patients (96%). In four patients, the oesophagus was not enhanced completely (Figure 3), due to peristaltic waves, but images were merged in an electro-anatomical map without difficulties. The breath-free MRA acquisition time, with double contrast agent administration using R-L phase encoding, was a mean of 7 s greater (range 5–12 s) than with the breath-hold MRA GRE-T1 breath-hold sequence without oral administration of contrast medium. No technical problems were encountered during first- or second-pass imaging.

## Discussion

Oesophageal injury is a rare but serious complication of RFCA. The oesophagus is positioned relative to the LA, adjacent to the right antra and ostia PV, as well as the posterior LA between the right and left PVs, or the left PV antra and ostia [12-15]. However, the position of the oesophagus is variable. Thus, the oesophagus could be at risk of thermal injury during RF ablation from virtually anywhere at the posterior left atrial endocardium or within the PV near the ostia, depending on the anatomy of the individual patient.

CTA and MRA are now the standard reference imaging techniques for LA and PVs. Volume rendering images obtained by these methods are merged on an electro-anatomical map generated with Carto™ (Biosense Webster, Diamond Bar, CA, USA) or NavX™ (St. Jude Medical, Sylmar, CA, USA) to guide catheter ablation. A report by Bahnson categorised the imaging methods to define the oesophagus-atrium relationship into real-time methods, including trans-oesophageal ultrasounds, nasogastric tube and barium paste, that yield images during RF-energy delivery; and non-real-time methods, including CT and MR, that yield static images generated prior to RF energy delivery.

Ultrasound is the only real-time imaging modality that allows visualisation of the extent or thickness of the oesophagus wall. However, this is an invasive imaging technique that requires operator experience. Placement of a nasogastric tube to allow oesophageal location mapping is associated with some risk of trauma and bleeding, particularly for anti-coagulation. Barium paste can be used to visualise the true width of the lumen for a few seconds, although information regarding oesophageal wall thickness is difficult to infer as only the lumen is visualised [16].

Real-time methods are more reliable for identification of oesophageal anatomy during RFCA than non-real-time methods. Kobza *et al.* performed a CT study using a common gastric tube as a marker the day before RFCA, and reported that the position of the oesophagus relative to the LA was dynamic and did not correspond to the position reported in the pre-procedural study [17]. Further, when the oesophageal position was defined by a pre-procedure CT scan with barium paste or by post-processing tagging and compared with the intra-procedure position defined using an electro-anatomical mapping system, in 85% of cases the position of the oesophagus corresponded to that identified with the CT scan performed <48 h prior [18,19]. This could be explained by a thin gastric tube placed on the side of the oesophagus. Our study demonstrates that a bolus better represents the true anatomical shape and size of the oesophagus.

Pavone *et al.* first performed a MR scan with barium paste and the MR contract agent, gadopentetate dimeglumine, to visualise the oesophageal lumen in patients with oesophageal stenosis [20]. MRA, besides providing detailed and complete imaging of the complex LA anatomy, does not expose the patient to ionising radiation [21]. Pollak *et al.* performed MRA with barium paste and gadopentetate dimeglumine in four candidates for RFCA; in one patient, the contrast agent passed to the stomach without enabling visualisation of the oesophagus [22]. A MRA scan is usually of longer duration than CT, and swallowing a contrast medium bolus at the correct time during a long breath-hold period could be difficult for the patient. In this study, using R-L phase encoding the patient could breathe freely and swallow the bolus during the MRA sequence without creating artefacts or affecting image quality. Although no technical problem was encountered with the first-pass MRA sequence, performing a second-pass immediately after the first MRA sequence could be useful for obtaining images suitable for anatomical assessment and calculating LA volume. With the second-pass MRA, we demonstrated that the contrast media and gel bolus rapidly pass through the oesophagus into the stomach, even in achalasic patients, without atrial wall artefacts, permitting fibrosis quantification associated with the likelihood of recurrent arrhythmia [23].

Allgayer *et al*. [24] reported that many MR protocols use atrial imaging to assess the anatomy and merge 3D images with an electro-anatomical map. However, only 3D-ECG-gated MRA allowed collection of the necessary anatomical information with optimal quality, although the acquisition time was >10 min. This study determined that the optimum solution for quality and patient tolerability was use of 3D MRA breath-hold non-ECG gated to assess the atrial anatomy necessary to merge the electro-anatomical map and 2D T2 axial images used to evaluate the theoretical position of the oesophagus. The MRA breath-free sequence used a comparable acquisition time to obtain 3D images of the atrium and oesophagus that could be merged into an electro-anatomical map.

Previous studies have reported that diluted gadolinium (Gd) complexes are safe for use as oral gastrointestinal contrast agents [25,26]. A Gd-BOPTA formulation was used in the present study because its signal intensity in T1-weighted images is greater than that of gadopentetate dimeglumine with increasing T1 relaxation times and dose. In human plasma, gadobenate dimeglumine is more effective in terms of shortening T1 relaxation times [27].

MR colonography is used in the gastrointestinal tract for faecal tagging because it is partly excreted in bile. Furthermore, the C-functionalised compound gadobenate dimeglumine exhibits high transmetallation towards metallic ions, such as Zn^2+^, and a long half-life dissociation in a strong hydrochloric acid (pH = 1) [28]. In addition, a study demonstrated that 99.2% of orally administered gadolinium contrast medium was excreted in the faeces and not absorbed [29]. In our study, we reported that the novel use of gadolinium-based contrast agent without barium paste allowed oesophageal enhancement during MRA. Furthermore, thickened gel water, which is often used when feeding weak and critical patients [30], could be used safely in patients suffering from a swallowing dysfunction and airway problems. Moreover, unlike barium, it is gluten, lactose, sugar and allergy free. Barium sulphate is also more costly than thickened water gel (2.18 € *vs.* ~0.60 € per patient), and at our institution, 1 ml of gadopentetate dimeglumine is priced similarly to gadobenate dimeglumine (5.7€ *vs*. 5.9€) [31].

## Conclusions

Oesophagus visualisation with oral administration of gadobenate dimeglumine MRA is a feasible technique without side effects and without significant increase in cost or examination time. Three-dimensional images obtained can be successfully merged during RFCA to characterise LA, PVs and oesophageal anatomy. Integration of oesophageal images into the electro-anatomical LA map could help to prevent RFCA injury to the oesophagus.

## Competing interest

The authors declare that they have no competing interests.

## Authors' contributions

AR, RF, FB: development of the technique and MRA imaging post-processing. MA, FF, FG: Interventional Cardiologists performing RFCA procedures and developing of electro-anatomical map integration. PF, GG, FG: Radiology and Cardiology Department Chiefs revising the manuscript. All authors read and approved the final manuscript.

## Acknowledgements

We would like to express our very great appreciation to Dr. Daniele Petrone and Dr. Davide Zamorani for their technical support and collaboration in this Study.
